# The Inclusion of Underrepresented Populations in Cardiovascular Genetics and Epidemiology

**DOI:** 10.3390/jcdd11020056

**Published:** 2024-02-05

**Authors:** Elias Chappell, Laura Arbour, Zachary Laksman

**Affiliations:** 1 Faculty of Medicine, University of British Columbia, Vancouver, BC V6T 1Z4, Canada; elichapp@student.ubc.ca; 2 Department of Medical Genetics, University of British Columbia, Vancouver, BC V6H 3N1, Canada; laura.arbour@ubc.ca; 3 Department of Medicine and the School of Biomedical Engineering, Centre for Heart Lung Innovation, University of British Columbia, Vancouver, BC V6T 1Z4, Canada

**Keywords:** cardiovascular epidemiology, cardiogenetics, underrepresented populations, diversity, genetics, genomics

## Abstract

Novel genetic risk markers have helped us to advance the field of cardiovascular epidemiology and refine our current understanding and risk stratification paradigms. The discovery and analysis of variants can help us to tailor prognostication and management. However, populations underrepresented in cardiovascular epidemiology and cardiogenetics research may experience inequities in care if prediction tools are not applicable to them clinically. Therefore, the purpose of this article is to outline the barriers that underrepresented populations can face in participating in genetics research, to describe the current efforts to diversify cardiogenetics research, and to outline strategies that researchers in cardiovascular epidemiology can implement to include underrepresented populations. Mistrust, a lack of diverse research teams, the improper use of sensitive biodata, and the constraints of genetic analyses are all barriers for including diverse populations in genetics studies. The current work is beginning to address the paucity of ethnically diverse genetics research and has already begun to shed light on the potential benefits of including underrepresented and diverse populations. Reducing barriers for individuals, utilizing community-driven research processes, adopting novel recruitment strategies, and pushing for organizational support for diverse genetics research are key steps that clinicians and researchers can take to develop equitable risk stratification tools and improve patient care.

## 1. The Current State of Cardiovascular Genetic Epidemiology for Common Traits and Diseases

Cardiovascular disease is the leading cause of death globally and comprises a group of diseases with a widespread impact [1]. While investigating the occurrence and distribution of disease, cardiovascular epidemiology also involves research into the determinants of clinical phenotypes. Among other factors, genetics play a significant role in cardiovascular disease pathophysiology and its clinical course [2,3]. Cardiogenetics is a broad and relatively novel field, which includes advancing cardiovascular epidemiology through the ability to identify individuals at risk of cardiac disease and guide their care using risk prediction tools [4,5,6].

Advancements in population-based cardiogenetics have primarily relied on the implementation of large genome-wide association studies (GWASs), which identify genetic variants associated with common traits and clinical phenotypes [4,5,6]. For example, coronary artery disease (CAD) GWASs have elucidated key associations between genetic loci, discovered new drug targets, and identified new genes important in CAD’s pathophysiology [5]. Each variant identified in a GWAS represents a small contribution to a phenotype; however, by aggregating multiple associated variants, one can better predict the occurrence of a trait. A polygenic risk score (PRS) is one such method for aggregation—formulated using the sum of risk alleles identified in GWASs for a given phenotype—that has widespread and clinically relevant use [7,8,9]. In cardiovascular epidemiology, PRS development is improving risk prediction and shaping clinical management in atrial fibrillation [10,11], CAD [12,13,14,15,16,17,18,19,20,21,22,23,24,25,26,27,28,29,30], cerebrovascular disease [31,32,33,34,35,36,37], hypertension [38,39,40,41,42,43,44,45], and heart failure [46,47,48,49].

While serving as an applicable risk prediction tool, a PRS’s utility and reliability is not uniform across individuals. For a given PRS derived from a GWAS composed of certain ancestries, its performance is best when applied to an individual of similar ancestry, and it lacks predictive power for different ancestries [50,51,52,53,54,55,56,57,58]. For example, Duncan et al. (2019) analyzed PRS studies from 2008 to 2017, finding that PRSs derived from European ancestry have poorer prediction in non-European populations [55]. Gola et al. (2020) illustrate that even within a European ancestry cohort, population-specific PRSs for CAD perform best in their respective subdivided population groups [58]. The poor transferability between populations occurs for various reasons, including variable degrees of associations comparing ancestries and differences in linkage disequilibrium that can reduce the effect sizes and subsequently decrease the predictive power [52].

An ongoing concern in genetic studies is a lack of cohort diversity. For example, the vast majority of GWASs (86%) are composed of participants of European ancestry [59]. The underrepresentation of certain groups, often minorities, is disproportionate to the overall population characteristics. The underrepresentation of diverse populations is reflected in cardiogenetics research [8], as well as cardiovascular trials [60,61,62]. GWASs thus far have poorly represented Hispanic/Latino, African, South Asian, East Asian populations [59,63,64], and especially Indigenous populations globally [55,65]. Inequities for these populations subsequently arise, as PRSs are not reliably able to predict risk, and therefore management cannot be personalized to these individuals. The study findings that highlight the underrepresentation of diverse populations in cardiovascular and genetics research are described further in Table 1.

While the current use of cardiogenetic methods in cardiovascular epidemiology is providing benefits to patients, inequities occur for those populations underrepresented in cardiovascular genetics research. Rectifying this gap is a matter of securing justice and equity for all individuals. At the same time, it is also imperative for the genomes of diverse populations to be studied for optimized clinical management, a reduction in health disparities, and understanding both human biology and our history [66]. Although cardiogenetics is a multi-faceted field, the importance of including diverse populations in GWASs and other genetics studies cannot be overstated. To underscore this, the purpose of this review is to answer: What are the barriers contributing to the underrepresentation of certain populations in cardiogenetics research? What efforts are researchers making to support diversity in cardiogenetics? What strategies can future cardiogenetics research use to include underrepresented populations?

## 2. Methodology

A broad review strategy was employed to answer the purpose questions of this study. A literature search of the following electronic bibliographic databases was conducted: MEDLINE, Embase, and ERIC. The search strategy was designed to capture the three separate avenues of inquiry and utilized the Boolean operators [AND] to separate subsections and [OR] to capture various terms within each subsection. Firstly, a search strategy containing “barriers”, “underrepresented populations”, “research”, “cardiovascular”, and “genetics” as subsections was developed using MeSH and field-designated search terms within each subsection. The subsections “cardiovascular” and “genetics” were alternately excluded from subsequent searches to maximize the identification of relevant work. A similar strategy was then developed with the replacement of “barriers” with the subsections “strategies” and “support” alternately searched. Lastly, a search strategy was developed containing the subsections “cardiovascular”, “genetics”, and “diversity” using MeSH and field-designated search terms. In addition, reference lists of key articles were screened to include pertinent studies not identified in this search strategy.

The inclusion criteria included: (1) publications from within the previous decade (1 January 2013 through 31 December 2023), unless this would exclude seminal works or a paucity of research was present, requiring more dated sources, (2) English language studies, (3) peer-reviewed studies.

**Figure 1:**
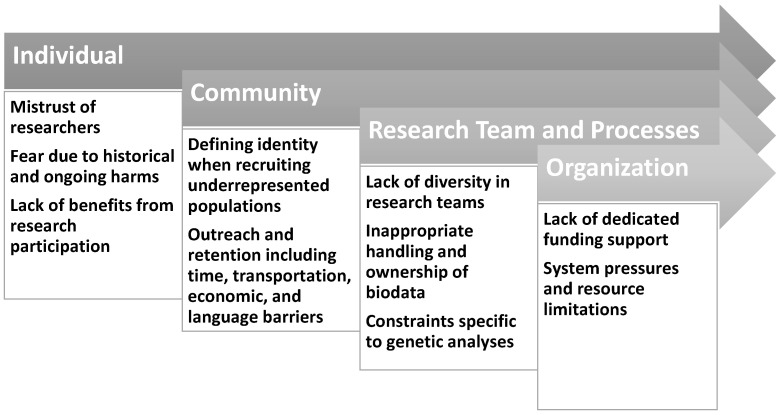
Barriers to inclusion of diverse populations in cardiovascular, genetics, and general research.

**Figure 2:**
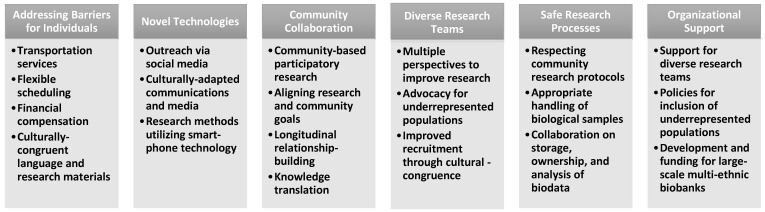
Strategies to support inclusion of underrepresented populations in cardiovascular and cardiogenetics research.

Both barriers to and strategies for the inclusion of underrepresented populations emerged organically through the results of this review, rather than being predetermined. The overarching themes were synthesized to formulate a natural organizational structure. Visual summaries of this review, detailed in Section 3 and Section 5, are found in Figure 1 and Figure 2, respectively.

## 3. Barriers to Inclusion for Diverse Populations

Difficulties in recruiting diverse population pools are a persistent problem in medical research [67,68,69]. Thus, in a similar vein, these obstacles are inherent in genetic and genomic research in addition to field-specific issues. Barriers occur at multiple points in the research process, presenting at the individual level, in communities, in research teams, and at the organizational level.

### 3.1. Mistrust

Starting at the individual level, one barrier commonly facing the recruitment of underrepresented populations is fear of harm and a lack of trust in researchers [70,71,72,73,74,75,76,77], a finding prominent in genetic research [77,78,79,80,81]. For minority individuals and communities that have experienced significant trauma due to unethical research practices, participating in research studies can be perceived as harmful and decrease participation [77,82,83]. A pertinent example is the Tuskegee Study of Untreated Syphilis, in which African American participants with HIV were observed and studied but not treated, which has resulted in long-standing mistrust of the healthcare system for African Americans [76,77]. Similarly, Indigenous populations have experienced harm through the medical system in various ways—one example being the forced sterilization of Native American Women via tubal ligations while under surgical anesthetic [84]. When considering genetic research, the Havasupai tribe in Arizona was subject to significant harm when researchers misused their DNA samples originally meant for type 2 diabetes research, instead using it for studies on highly stigmatized topics, including schizophrenia and migration theory [85]. Another example is the exploitation of blood samples given by the Nuu-chah-nulth tribe in British Columbia, Canada, for the investigation of rheumatoid arthritis prevalent in their community, producing no findings; the researchers instead conducted different research with the samples, including work on the infectious spread of viruses via intravenous drug use [86]. Given these occurrences, minority populations have valid concerns in participating in research projects that may harm them by increasing stigma, with the potential for discrimination.

In addition to fearing harm through the research process, many underrepresented populations have experienced little benefit from participating in research. Historically, researchers have performed research on minority communities rather than with them [87,88]. Extracting data from populations without proper consent and consultation may prevent relevant research important to communities from being carried out [85,86]. Given previous and ongoing research work that benefits the investigators and may harm the minority participants, communities may not engage with contemporary research efforts and subsequently become underrepresented in data sets.

### 3.2. Identifying and Reaching Populations

Identifying underrepresented populations and ensuring outreach is targeted effectively is another barrier in research recruitment. Self-identification is typically used to collect ethnic data; however, the categorization researchers or participants choose may reflect socially defined ethnicity rather than the ancestral population targeted, where the latter is particularly relevant in genetics research [70,89]. Additionally, underrepresented populations may be genetically and ethnically heterogeneous, as is the case between different Indigenous communities [65]. Therefore, broad categorizations may not reflect clinically relevant cohorts and obscure important associations [65]. In addition to identification, out-reach to specific target populations can also come with difficulties. Underrepresented populations may face obstacles in accessing healthcare services for a variety of reasons—including time constraints [72,73,74,75], transportation difficulties [71,72,73], and financial burden [71,72]—decreasing their opportunities to participate in research endeavors and ability to attend research appointments. Differences in health literacy, language, and exposure to the healthcare system can also lead to miscommunication and a lack of opportunity to meaningfully engage in research [72].

### 3.3. Organizational Constraints: Diversity and Funding

A lack of diverse representation in research teams is another barrier to the inclusion of underrepresented populations [65,70]. The current diversity in the medical research workforce has improved compared to previous measures but is still disproportionate to the general population [90,91,92]. Without input from population stakeholders, the research goals may be misaligned from the participant goals, leading to dissatisfaction and a lack of engagement in research. Health literacy and language barriers are other factors not easily overcome without the representation of culturally congruent and linguistically appropriate team members [71,72]. Difficulties in retaining minority researchers center around cultural safety and support in ensuring diverse teams are formed, which requires dedicated policies and funding [93]. Similarly, system pressures and resource limitations may make the intensive strategies necessary to recruit underrepresented populations difficult to implement [94]. Ultimately, poor recruitment and retention of minority researchers, coupled with a need for significant institutional funding, are key barriers to including diverse populations in genetics research.

### 3.4. Appropriate Handling of Biodata

The collection, analysis, storage, and ownership of biodata is particularly relevant in genetics research. Some cultures view biological samples as incredibly important pieces of information, representing extensions of themselves and connections with relatives and belief systems [83,95]. As such, a significant amount of trust is needed to provide biodata to researchers; considering the previous and ongoing harm that research has caused, an individual or community may feel the risk is too great. Questions of ownership and how the results of research are disseminated is another pertinent issue when including underrepresented populations in genetics research [65,96,97,98].

### 3.5. Constraints of Genetic Analysis

As Bentley et al. (2017) describe, the propensity for research focusing on European ancestry cohorts can be attributed to their greater relative availability in large open-access datasets. Given that a genomic study’s validity is evaluated with sample size being a key factor in revealing associations, researchers may find it prudent to utilize these cohorts over others [66]. A higher level of linkage disequilibrium in European ancestries relative to other cohorts can make analysis in European populations more efficient due to the discovery of more variant associations [66]. An additional barrier is the higher genetic diversity in certain populations such as African ancestries compared to European, making the discovery of variant associations more difficult and analysis more complicated without subdivision [99]. The available technologies may also not be suitable for analysis in genetically diverse cohorts given their lack of inclusion in the current models—for example, the genomic reference panel derived from the 1000 Genomes Project dataset does not represent many South Asian and African population genomes [100]. The addition of diverse ancestries allows for the discovery of risk variants that would be otherwise unearthed. This is also true for single-gene disorders such as hypercholesterolemia, cardiomyopathy, and inherited arrhythmias [101,102], where large multi-gene panels are used for diagnoses. The knowledge of which variants are common or rare in a population improves the ability to confirm a precise diagnosis. Endeavors such as the “Silent Genomes Project” in Canada [103] and the “Aotearoa Variome” in New Zealand [104] are building Indigenous background variant databases to further genetics research and care for these chronically underserved and heterogeneous communities [65]. Other multi-ethnic platforms, such as the “All of Us” research program [105], Million Veteran Program [106], the UK Biobank [107], and Biobank Japan [108], are providing researchers with a more diverse pool to draw from for equitable genomic research. The evidence shows that dedicated research programs working toward diversity in genetic research can improve the representation of historically underrepresented populations [109].

## 4. Increasing Diversity in Cardiogenetics Studies

Despite the numerous barriers present to including diverse populations in research endeavors, early diversification efforts to include underrepresented populations in cardiogenetics studies are underway. For cardiogenetic prediction tools to be calculated, large genetic databases must be available for a given population. Recognizing this, a major step in increasing representation has been addressing the paucity of diverse genetic biobanks. For example, Legget et al. (2021) detail a large-scale project, the Multi-Ethnic New Zealand Study of Acute Coronary Syndromes (MENZACS), which comprises a large diverse biobank for future prospective study of the genetic factors influencing ACS [110]—the represented populations include Māori, Pacific, Indian, and NZ European.

As further genetic data from underrepresented populations are collected, analyses can draw on them and incorporate them into future efforts toward PRS development. As has been detailed previously, PRSs created from primarily European ancestry cohorts tend to perform poorly in non-European populations. The work being performed now illustrates the strength that diverse genetic backgrounds can provide to PRS performance. Martin et al. (2019) demonstrated that prediction tools from ancestry-matched GWAS summary statistics had an improved accuracy in predicting anthropometric measures and disease endpoints [52]. Wojcik et al. (2019) discuss the Population Architecture using Genomics and Epidemiology (PAGE) study and its efforts in conducting genetic epidemiological research in diverse populations; the authors found evidence of a heterogeneous effect size across different ancestries for BMI and height and demonstrated how the fine mapping of gene loci to diverse populations increases association discovery [111]. Mahajan et al. (2022) demonstrated how utilizing a meta-analysis of multi-ancestry GWAS studies allowed for greater transferability of predicting type 2 diabetes across various populations. The authors additionally elucidated the specific genetic mechanisms discovered via fine-mapping that provided the basis for functional investigations, made possible by a greater population diversity [112].

There is also evidence that the incorporation of diverse ancestral backgrounds is being considered in cardiovascular epidemiology as researchers begin to address this gap and these unmet needs. Kullo and Dikilitas (2020) describe a framework for using coronary heart disease (CHD) PRSs in a specific population, entailing the determination of where an individual’s score falls in an ancestry-matched distribution to categorize them into low-, medium-, and high-risk groups [113]. This strategy allows for contextualized PRSs that better guide an individual’s cardiac risk assessment even if their ancestry is relatively underrepresented in GWASs. Wang et al. (2020) illustrated the derivation and validation of an ancestry-specific CHD PRS for South Asians from a larger majority-European GWAS and were able to demonstrate improved predictions and the ability to risk-stratify individuals [114]. Koyama et al. (2020) provided evidence of a CAD PRS derived from a trans-ancestry meta-analysis outperforming population-matched Japanese and English PRSs [115]. Kurniansyah et al. (2022) similarly developed a PRS based on multi-ethnic hypertension GWAS data, which had a good predictive performance for incident hypertension at follow-up [41]. Tcheandjieu et al. (2022) drew on the Million Veteran Program to report a new GWAS of CAD comprising primarily White, Black, and Hispanic participant ancestries. In addition to identifying numerous novel loci of interest and providing evidence for the disease mechanisms in CAD, the authors reiterated how the current PRSs, derived from European ancestry populations, have poor transferability to Black populations—with a new PRS derived from their diverse GWAS, the risk prediction was improved across all populations [30]. The authors highlight the importance of data-gathering in non-White populations and the refinement of analyses to reduce the PRS performance variability between ethnic populations [30]. While some studies have shown the derivation of PRSs from diverse populations does not outperform population-specific PRSs [30,112], overall, the majority of the literature to date continues to indicate the value of including underrepresented populations.

## 5. Strategies for the Inclusion of Underrepresented Populations

Including ancestrally diverse populations in cardiogenetics research improves the strength and validity of the reported outcomes, while also serving minority populations facing inequitable care. Addressing the gaps underrepresented populations face in healthcare is an incredibly important undertaking, but not without its challenges. Discussion of the key strategies that can be employed by clinicians and researchers can aid in future research endeavors.

### 5.1. Addressing Barriers for Individuals

At the level of the individual, common barriers to those from underrepresented populations engaging with research efforts include time constraints, a lack of resources, and a mismatch between language and health literacy [71,72,73,88]. To counteract these obstacles, recruitment efforts should seek to alleviate individual burdens through appropriate funding and support. For example, Ejiogu et al. (2011) developed a multi-pronged approach to recruiting a socioeconomically diverse cohort of African American and non-Hispanic White participants for a longitudinal age-related study on health disparities. The researchers addressed transportation barriers using mobile data collection centers and free transportation, time and economic constraints using flexible scheduling and financial compensation, and differences in health literacy and language using a culturally congruent and diverse research team [73]. Transportation interventions in particular have been well studied, with evidence suggesting no-show rates for appointments decreased with transportation aid [116,117]. Similarly, a financial support program for cancer clinical trial participation showed an increase in enrollment after its implementation [118]. Frameworks for the recruitment of minority populations support the application of culturally congruent research materials and staff to mitigate differences in language and health literacy [119,120]. These interventions can improve the trust and comfort of potential participants and enhance enrollment [121,122].

### 5.2. Novel Technological Strategies

Outreach endeavors for underrepresented populations that are hindered by opportunity or sociocultural divides may benefit from the utilization of novel technological strategies and mediums [119,120,123]. For example, outreach via social media can be an effective method of participant recruitment in underrepresented populations, especially when communications are culturally adapted [124,125,126]. While not all studies show social media as their most effective outreach strategy, these methods still provide a viable and cost-effective method as part of a well-rounded recruitment approach [127]. Brewer et al. (2018) illustrate how a smart-phone app paired with a community cardiovascular health program can enhance success in recruitment and the utilization of evidence-based health interventions [123]. While technological recruitment strategies offer a promising route, care should be taken when implementing these novel multi-media strategies in underrepresented populations, as the ethical considerations are still nascent, under examination, and without specific oversight or official guidelines [128].

### 5.3. Collaborating with Communities

Given the substantial evidence of harm and a perceived lack of benefit for underrepresented populations in research [77,83,85,86,87], it is necessary, and beneficial, that research be formulated with community-specific guidance from the beginning [129,130,131,132]. Community-based participatory research (CBPR) is a widely used and effective methodology to ensure benefit to the community and meaningful research goals [133]. Collaboration, shared decision-making, and shared ownership of research materials and products are key values of CBPR projects [133]. Cultural differences between participants and researchers may account for underrepresentation in genetics research, where a minority population’s motivation for research engagement may, for example, be focused on the benefit to their community rather than publication [134]—this makes CBPR values incredibly important to ensure participant engagement. Trust remains a hallmark of effective collaboration with a community; conceptual models, such as a circle of trust, can help guide engagement efforts that include unrepresented and marginalized communities [135]. Communication and relationship-building should be approached in a longitudinal fashion according to formal and informal connections [96,120,136]. Collaborating with communities prior to any research activity is key for success, as it can guide design and provide valuable new ideas [130]. Arbour et al. (2008) demonstrated cardiogenetics research driven by CBPR principles with a Canadian First Nations community, initiating research to investigate the high rates of Long QT syndrome in their community and its genetic basis [137]. Ensuring research activities and products are appropriately disseminated to participants and ongoing regular communication with stakeholders are other essential aspects of CBPR [96,130]. The knowledge translation should be enacted in an understandable and transparent manner to ensure meaningful community involvement [138]. Further recommendations drawing from CBPR principles have been formulated under a genetics- and genomics-specific research lens to guide future work [94,139].

### 5.4. Promoting Diverse Research Teams

Promoting diversity within research teams is a well-evidenced strategy to improve research outcomes and reach underrepresented populations. The literature shows that diverse groups increase creativity, the impact of research, and the output of high-quality work that can benefit populations experiencing inequities [140,141,142,143,144]. Blanchard et al. (2017) note how having an Indigenous researcher’s perspective allowed for a more culturally appropriate research design and decreased potential harm in a qualitative study of perceptions on genetic ancestry testing [70]. Promoting diverse teams using community capacity-building is a strategy that can produce competent professionals while aligning the research goals with a population’s needs [130]. Ultimately, having a diverse team that reflects the culture and languages of prospective study populations can decrease mistrust and promote recruitment [70,73,120,121,122,136]. Recruiting and retaining a diverse workforce in genetics research is imperative to future work; this fact is highlighted by researcher recommendations [71,94,119] and key organizations such as the National Human Genome Research Initiative including workforce diversity in their strategic vision and recommendations [145].

### 5.5. Safe Research Processes

Research protocols and practices involving underrepresented populations should have safety as a core value in their formulation. Respecting community research protocols and processes is a key step researchers can take in ensuring the safety of research performed with underrepresented populations [70]. For example, many Indigenous communities have their own institutional review boards and regulatory bodies to safeguard their members against potentially harmful projects [130]. Safety is particularly important in the case of genetics research, given the use of more sensitive data consisting of biological samples. Given the previous harms enacted via geneticists in the past [85,86], and the cultural significance of tissue samples inherent to certain populations [83,95], biodata should be collected, stored, and analyzed under community-driven protocols and wishes [96].

### 5.6. Support for Diverse Genetics Studies

Calls for dedicated recruitment of diverse cohorts for genetics research are ongoing given the continuing paucity of underrepresented populations in clinically impactful studies [93,94,146]. Several large-scale endeavors are responding to these recommendations, including the development of multi-ethnic biobanks that can be used for genetics research purposes [103,104,105,106,107,108,110]. Given the sheer number of data points needed for well-powered genetics studies, the cost–benefit in the case of large-scale biobank development is evident [147]. Funding is paramount in genetics research, and financial support for genetics studies in underrepresented populations illustrates the progress that can be made [61]. Simultaneously, care and culturally appropriate methods should be implemented in the building of such databases to ensure harm is not perpetuated and that population-specific contexts and goals are honoured [146,147]. While the addition of underrepresented populations to GWASs and biobank studies progresses, novel strategies in the cardiogenetic analysis of multi-ethnic data sets already available should be utilized and developed to begin increasing health and genetic care equity for underrepresented populations [113,114,148].

## 6. Study Limitations

Given the nascency of cardiogenetics as a field, there was a relative paucity of published works available to assess and synthesize. We therefore sought to highlight pertinent issues pertaining to underrepresented populations as they are developing in cardiovascular epidemiology and genetics. However, with increasing available evidence, scoping and systematic review methodologies should be implemented to assess and strengthen strategies for inclusion. Limiting our searches to English language publications may have resulted in missing perspectives and underestimating representation.

## 7. Conclusions

Genetic advancements in cardiovascular epidemiology must be accompanied by the equitable implementation of novel care modalities. Establishing that certain populations are underrepresented in cardiovascular and genetics research, this review found that mistrust, difficulties in reaching certain populations, a lack of research team diversity, the inappropriate handling of biodata, and constraints particular to genetic analysis all present potential barriers for increasing diversity in genetics studies. Despite these obstacles, the development of large multi-ethnic biobanks and the incorporation of diverse populations into cardiogenetics research is promising in terms of the future benefits to all those impacted by cardiovascular disease. The strategies cardiogenetic researchers can implement to include diverse populations involve reducing individual barriers, utilizing community-driven research processes, adopting novel technologies and methods in recruitment, and advocating for organizational support and funding.

## 8. Future Directions

Future work should aim to incorporate the strategies outlined in this review to support and improve future cardiovascular epidemiology and genetics studies. Future directions in cardiogenetics research can include the development of guidelines and criteria in using PRSs within a given ancestry group to ensure reliable performance. Future cardiovascular epidemiology work should additionally assess the progress made in including underrepresented populations using scoping and systematic reviews. Overall, creating equitable risk prediction tools that benefit all patients with specialized care is an important and necessary step toward advancing cardiovascular epidemiology research and clinical outcomes.

## Figures and Tables

**Table 1:** Key studies illustrating the underrepresentation of diverse populations in genetics and cardiovascular research.

Authors	Study Type	Key Findings
Azzopardi et al. [61]	Systematic Review	The authors performed a systematic review of cardiometabolic trials between 2011 and 2020, finding a low overall proportion of Asian participants at 8.3% with a marginal increase over time. Regional enrollment was disproportionate when compared to burden of disease between regions.
Duncan et al. [55]	Review	Analyzing PRS studies from 2008 to 2017, the authors found 67% of studies were derived from European ancestry cohorts, with only 3.8% from African, Hispanic, or Indigenous participant cohorts.
Fatumo et al. [59]	Review	The authors illustrate how the vast majority of GWASs, 86%, are derived from European ancestry cohorts as of 2021, which is an increase from 81% in 2016. Studies with multi-ancestry cohorts have increased slightly, but the proportion of GWASs including underrepresented populations has plateaued or decreased since 2016. Underrepresented populations include East Asian, South Asian, African, Hispanic/Latino, Greater Middle Eastern, Oceanic, and Other (including Indigenous populations).
Landry et al. [64]	Review	The authors analyzed data from two public genomic databases, finding the majority of genetic studies were based on European ancestry cohorts, with Asian populations comprising the next largest proportion and the rest underrepresented minority groups (often <5%). For some diseases, no GWASs of underrepresented populations were present. Cardiovascular disease studies represented minorities slightly better, with 12% of GWASs from underrepresented populations and 68% from European.
Popejoy & Fullerton [63]	Review	The authors analyze published GWASs, finding 81% are derived from European ancestry cohorts in 2016 compared to 96% in 2009. The authors note that this improvement in diversity has largely been driven by the inclusion of East Asian studies.
Phulka et al. [8]	Scoping Review	Focusing on the clinical utility of cardiometabolic PRSs, the authors found limited ancestral diversity in PRSs with the majority being derived from European ancestry cohorts. For example, 29 of 37 published PRSs for coronary heart disease were developed from European ancestry cohorts.
Vilcant et al. [62]	Review	The authors reviewed landmark cardiovascular trials between 1986 and 2019, finding that percentages of non-White participants did not significantly change over time, with an average of approximately 20%. The findings indicate a lack of improvement in cardiovascular trial participant diversity.
Zhang et al. [60]	Systematic Review	The authors reviewed major cardiovascular RCTs published between 1997 and 2010, finding a median enrollment rate of 86% White participants. White participant enrollment was overrepresented in CAD clinical trials when compared to population prevalence, and African Americans were underrepresented.
